# Safety evaluation of conditionally immortalized cells for renal replacement therapy

**DOI:** 10.18632/oncotarget.27152

**Published:** 2019-09-03

**Authors:** Milos Mihajlovic, Sam Hariri, Koen C.G. Westphal, Manoe J. Janssen, Miriam J. Oost, Laura Bongiovanni, Lambertus P. van den Heuvel, Alain de Bruin, Luuk B. Hilbrands, Rosalinde Masereeuw

**Affiliations:** ^1^ Division of Pharmacology, Utrecht Institute for Pharmaceutical Sciences, Utrecht University, Utrecht, The Netherlands; ^2^ Dutch Molecular Pathology Centre, Department of Pathobiology, Faculty of Veterinary Medicine, Utrecht University, Utrecht, The Netherlands; ^3^ Department of Pediatric Nephrology, Radboud University Medical Center, Nijmegen, The Netherlands; ^4^ Department of Nephrology, Radboud University Medical Center, Nijmegen, The Netherlands

**Keywords:** bioartificial kidney, cell therapy safety, conditionally immortalized proximal tubule epithelial cells, tumorigenicity, viral integration

## Abstract

End-stage kidney disease represents irreversible kidney failure. Dialysis and transplantation, two main treatment options currently available, present various drawbacks and complications. Innovative cell-based therapies, such as a bioartificial kidney, have not reached the clinic yet, mostly due to safety and/or functional issues. Here, we assessed the safety of conditionally immortalized proximal tubule epithelial cells (ciPTECs) for bioartificial kidney application, by using *in vitro* assays and athymic nude rats. We demonstrate that these cells do not possess key properties of oncogenically transformed cells, including anchorage-independent growth, lack of contact inhibition and apoptosis-resistance. In late-passage cells we did observe complex chromosomal abnormalities favoring near-tetraploidy, indicating chromosomal instability. However, time-lapse imaging of ciPTEC-OAT1, confined to a 3D extracellular matrix (ECM)-based environment, revealed that the cells were largely non-invasive. Furthermore, we determined the viral integration sites of SV40 Large T antigen (SV40T), human telomerase (hTERT) and OAT1 (SLC22A6), the transgenes used for immortalization and cell function enhancement. All integrations sites were found to be located in the intronic regions of endogenous genes. Among these genes, early endosome antigen 1 (EEA1) involved in endocytosis, and BCL2 Like 1 (BCL2L1) known for its role in regulating apoptosis, were identified. Nevertheless, both gene products appeared to be functionally intact. Finally, after subcutaneous injection in athymic nude rats we show that ciPTEC-OAT1 lack tumorigenic and oncogenic effects *in vivo*, confirming the *in vitro* findings. Taken together, this study lays an important foundation towards bioartificial kidney (BAK) development by confirming the safety of the cell line intended for incorporation.

## INTRODUCTION

End-stage kidney disease (ESKD) represents irreversible kidney failure through a variety of causes. Ageing of the population, with frequently occurring diabetes, atherosclerotic vascular disease, and hypertension, is predominantly responsible for an increasing prevalence of ESKD [1–3]. Despite the large socioeconomic impact of ESKD [4], innovative novel therapies have thus far failed to reach the clinic. Recognition of the problems related to currently available treatments, has spurred the development of novel approaches of which cell-based systems, also known as bioartificial kidney (BAK), that seek to replicate the kidney’s function through the integration of proximal tubule cells are of promise [5].

One of the crucial issues to take into consideration when developing a BAK is the sufficient availability of suitable cells. Human primary proximal tubule epithelial cells (PTEC) have a limited life span *in vitro* and presenting risks, such as functional changes occurring upon culturing as well as dedifferentiation and senescence of cells [6, 7]. Several studies have therefore focused on animal cells [8–10], or cell lines [11–15]. Issues related to the use of animal-derived cells in BAK are safety concerns compromising approval for clinical application and species differences in cell behaviour.

To overcome these limitations, we employed human urine-derived PTEC that were conditionally immortalized using the essential catalytic subunit of human telomerase (hTERT) and a temperature-sensitive mutant U19tsA58 of SV40 large T antigen (SV40T), creating conditionally immortalized PTEC (ciPTEC) [16]. Due to the expression of temperature-sensitive SV40T, cells can be expanded at permissive temperature of 33° C and differentiated into mature cells at non-permissive temperature of 37° C [16–18]. While hTERT acts by stabilizing telomeres, thus preventing the occurrence of replicative senescence [19], SV40T involves the activation of E2F-mediated transcription through binding with Rb-E2F complex, as well as the inhibition of p53 [20, 21]. The cell line has been thoroughly characterized over the years [16, 22–25], but the absence of the physiologically important organic anion transporter 1 (OAT1) protein led us to modify ciPTEC further by an overexpression of the transporter [26]. With this cell line we demonstrated the capacity of an efficient removal of uremic toxins when cells are cultured on hollow fiber membranes (HFM), thereby creating fully functional kidney tubules [27]. In addition to the proven lack of ciPTEC allostimulatory potential *in vitro* [28], a successful upscaling of the biofunctionalized HFM with tight epithelial monolayers and cell function has been achieved, encouraging further efforts towards the BAK development [29].

Eyeing possible clinical applications of ciPTEC, a thorough safety evaluation is warranted to exclude any risks related to oncogenesis and tumorigenesis [30]. Even though SV40T mediated inhibition of p53 and Rb pathways or telomere length maintenance by hTERT are not sufficient to induce oncogenic transformation, various parameters related to cell growth and proliferation, apoptosis and migration have to be examined [31–35]. Given that the transgenes were introduced by retroviral and lentiviral transductions, which can be oncogenic through insertional mutagenesis [36–38], we evaluated if the transgenes had disrupted proto-oncogenes or important genes required for PTEC function. Finally, we evaluated cell transforming properties and tumorigenic potential *in vivo* to gain more insight into safety and suitability of these cells for applications in renal replacement therapies.

## RESULTS

### The proliferative capacity and apoptosis resistance of ciPTEC in relation to SV40T expression

**Figure 1:**
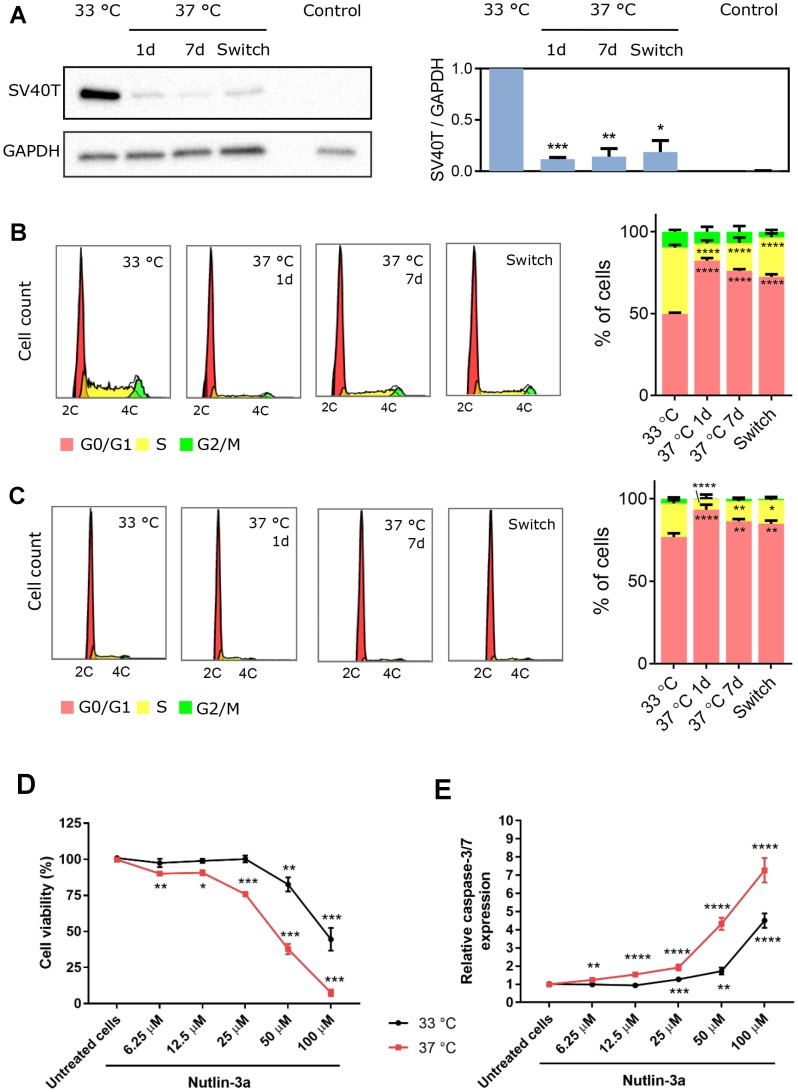
Temperature-dependent effect of SV40T expression on ciPTEC-OAT1 proliferation and apoptosis-sensitivity. (**A**) Western blot analysis of SV40T levels in ciPTEC-OAT1 cultured at the permissive (33° C) temperature and the non-permissive (37° C) temperature for 1 day, 7 days or 7 days followed by a 4 h incubation at 33° C (switch). Intensity of the bands was normalized to GAPDH and quantification is depicted in the bar graph. Human kidney tissue protein sample served as control. Representative histograms and analysis of cell cycle distribution of ciPTEC-OAT1 cultured at (**B**) subconfluent and (**C**) confluent levels at 33° C and 37° C for 1 day, 7 days or 7 days followed by 4 h at 33° C (switch). (**D**) Cell viability analysis and (**E**) caspase-3/7 expression in ciPTEC-OAT1 cultured at 33° C and 37° C and exposed to increasing concentrations of nutlin-3a for 24 h. All values are expressed as the mean ± SEM of three independent experiments performed in triplicate. ^*^ *p* < 0.05, ^**^ *p* < 0.01, ^***^ *p* < 0.001 (unpaired two-tailed Student’s *t*-test and one-way ANOVA followed by Dunnett’s multiple comparison test).

As expected [16], the expression of SV40T was abundant at permissive temperature (33° C) but went down (90% reduction) within one day of culturing at a non-permissive temperature (37° C) and remained low for up to 7 days (Figure 1A). Clinical studies indicated that cooling the dialysate down to 35° C can be beneficial as it may enhance the patient’s hemodynamic stability by preventing intradialytic hypotension [39, 40]. A transient drop in temperature (4 h, 33° C), however, did not result in an increase in SV40T expression compared to cells maintained at 37° C for 7 days (Figure 1A).

In a subconfluent state a higher proportion of cells was found in S-phase at the permissive temperature (Figure 1B; 40.5% ± 1.7%) compared to 37° C for 1 day (10.5% ± 2.0%) or 7 days (17.0% ± 3.4%), confirming that SV40T protein expression is directly related to cell proliferation. At full confluency (Figure 1C), the same trend was observed with a higher proportion of proliferating cells at the permissive (20.4% ± 1.9%) compared to the non-permissive temperature (6.3% ± 6.9% after 1 day at 37° C and 12.1% ± 1.5% after 7 days at 37° C).

Following 24 h of exposure to nutlin-3a to trigger p53-mediated apoptosis, one of the main targets of SV40T [20], matured cells expressing lower levels of SV40T displayed higher sensitivity to nutlin-3a compared to cells cultured at permissive temperature. Indeed, cells at 33° C were resistant to nutlin-3a-induced cell death, whereas cells at 37° C showed reduced cell viability and higher expression of caspase-3/7 even at lower nutlin-3a concentrations (Figure 1D–1E).

### CiPTEC-OAT1 obey the rule of contact-inhibition

**Figure 2:**
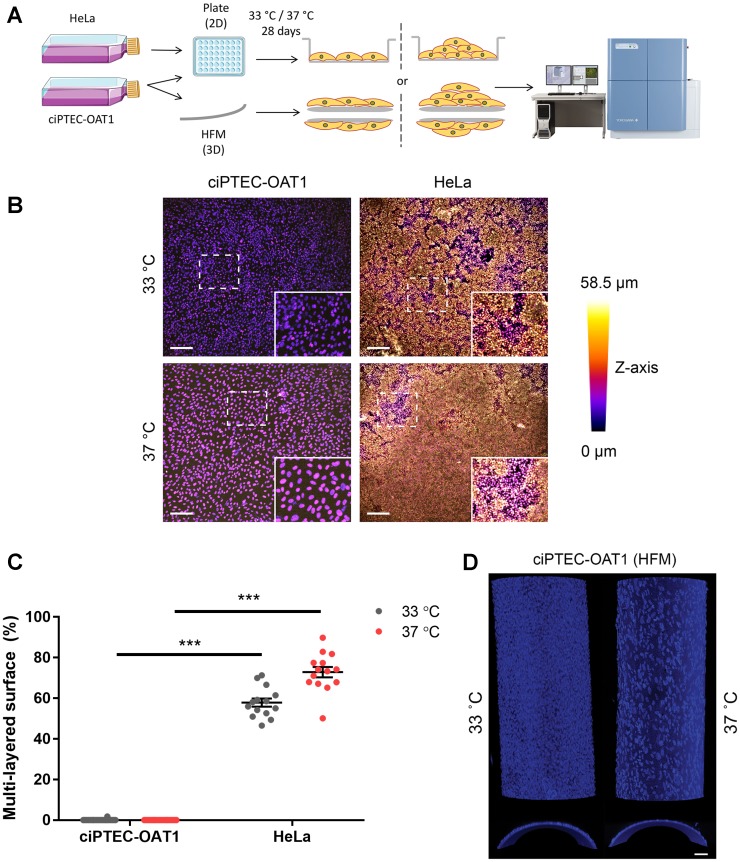
Contact inhibition in ciPTEC-OAT1. (**A**) Schematic diagram of focus formation assay. CiPTEC-OAT1 were cultured in 2D (96-well microplate) and 3D (hollow fiber membranes; HFM) for 28 days at 33° C and 37° C. HeLa cells were cultured in 2D in same conditions. Foci (multi-layered growth) formation was detected by nuclear staining and confocal imaging and (**B**) representative depth-coded images of nuclei-stained ciPTEC-OAT1 and HeLa cells after 28 days of culture at both permissive and non-permissive temperature are shown. Scale bars denote 200 μm in the original image and 100 μm in the zoom-in. (**C**) Quantification of the surface area covered by multi-layered proliferation). ND = not detected. (**D**) Representative confocal images of nuclei stained ciPTEC-OAT1 cultured on double-coated HFM at 33° C and 37° C, x-y confocal planes on the upper part and y-z confocal planes on the bottom part. Images taken with 10× magnification. Scale bar: 50 μm. Values are expressed as the mean ± SEM of three independent experiments performed in triplicate. ^***^ *p* < 0.001 (unpaired two-tailed Student’s *t*-test).

Further, ciPTEC-OAT1 growth did not extend beyond a confluent epithelial monolayer regardless of permissive or non-permissive temperature (Figure 2A), indicating contact inhibition. In contrast, HeLa cells presented multi-layered growth with >55% of growth surface covered with multiple cell layers when cultured at 33° C, and ~70% of multi-layered cell growth at 37° C (Figure 2B–2C). When ciPTEC-OAT1 were cultured on HFM, multi-layered growth was also not observed at both temperatures (Figure 2D).

### CiPTEC-OAT1 require anchorage for proliferation

**Figure 3:**
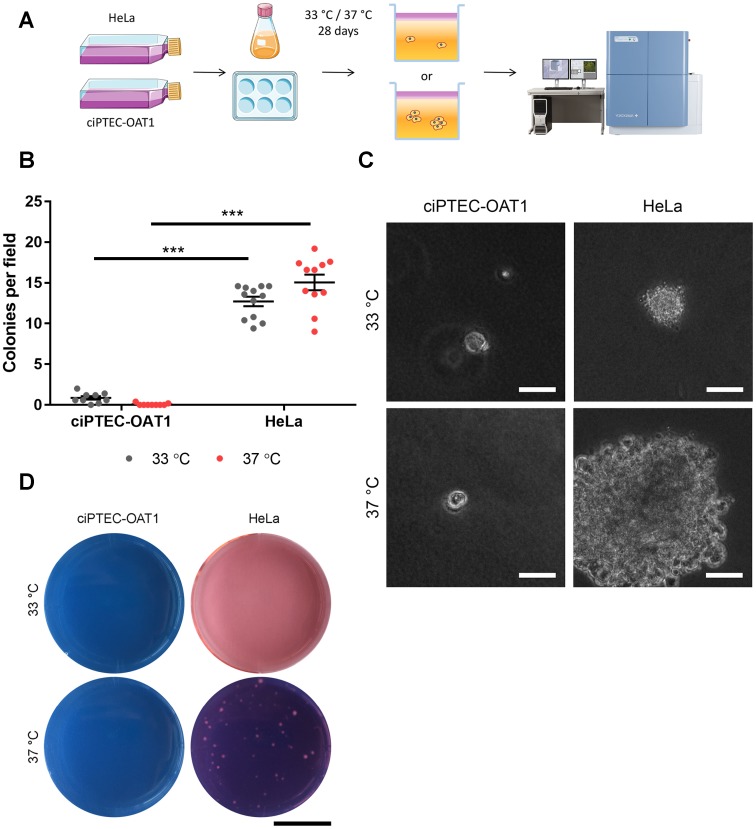
Anchorage-independent growth at permissive and non-permissive temperatures. (**A**) Schematic diagram of soft agar assay. Single ciPTEC-OAT1 and HeLa cells were seeded in agarose-containing medium (0.3% (w/v)) and incubated for 28 days at either 33° C or 37° C. Cell growth and colony formation was detected by confocal imaging. (**B**) Quantification of colonies detected for ciPTEC-OAT1 and HeLa cells presented as number of colonies per field. (**C**) Representative microscopic pictures of cell colonies formed by ciPTEC-OAT1 HeLa cells after 28 days culture at 33° C and 37° C. Scale bars denote 50 μm. (**D**) Representative macroscopic pictures of ciPTEC-OAT1 and HeLa cell colonies. Scale bar denotes 1 cm. Values are expressed as the mean ± SEM of three independent experiments performed in triplicate. ^***^ *p* < 0.001 (unpaired two-tailed Student’s *t*-test).

Anchorage-independent growth is a hallmark of cancer cells [41, 42], and it is assessed using the soft agar assay (Figure 3A). Single cells were encapsulated in semi-solid agarose medium thereby creating an environment lacking anchorage means (i.e. cell-cell and cell-extracellular matrix (ECM) interactions). After 4 weeks of culture, sporadic colony formation was observed for ciPTEC-OAT1 (0.87 ± 0.21 colonies per field (CPF)) compared to control HeLa cells (12.70 ± 0.58 CPF). Similarly, colonies were absent in cultures of mature ciPTEC-OAT1 (0.07 ± 0.05 CPF) while abundantly present in cultures of HeLa cells (15.10 ± 0.95 CPF) (Figure 3B). Qualitatively, colonies incidentally observed in ciPTEC-OAT1 cultures were much smaller than in HeLa cell cultures (Figure 3C), as also illustrated by the macroscopic images (Figure 3D), where only HeLa cells formed colonies at 37° C large enough to be visible by eye.

### CiPTEC-OAT1 are predominantly non-invasive

**Figure 4:**
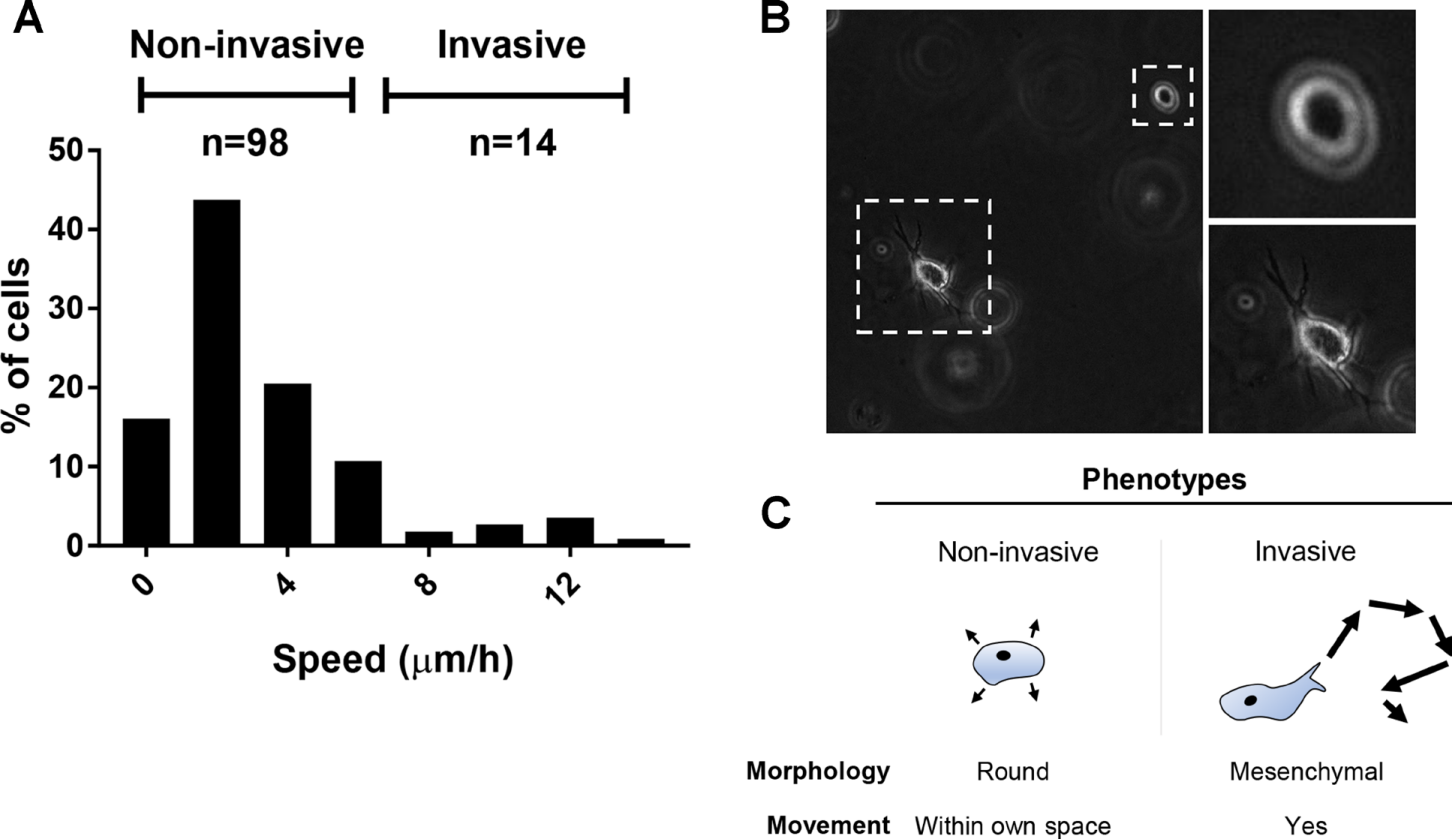
Migration and invasive potential of ciPTEC-OAT1. (**A**) Histogram showing the average speed (μm/h) at which the cells moved through Matrigel™ basement membrane matrix during the 24 h time-lapse imaging. Obtained values arose from 112 cells analyzed from two independent experiments. (**B**) Representative pictures showing morphological differences between the invasive and non-invasive phenotypes (20× magnification). (**C**) Invasive cells displayed mesenchymal cell-like movement, while non-invasive cells were round and static, wobbled only within their own space.

A cell-tracking experiment in growth factor reduced Matrigel™ was performed to model invasive and metastatic capacity of matured ciPTEC-OAT1 *in vitro*. During a 24 h time-lapse, the majority of cells (89.0%) did not migrate and remained non-invasive (Figure 4A, Supplementary Figure 1). However, a small population was able to migrate through the ECM. Morphologically, these cells showed mesenchymal cell-like features and moved accordingly with a speed higher than 6 μm/h, whereas non-invading cells remained round-shaped with minimal movement within their own space at a speed below 6 μm/h (Figure 4B, 4C).

### Transgene integration sites and genomic stability

**Figure 5:**
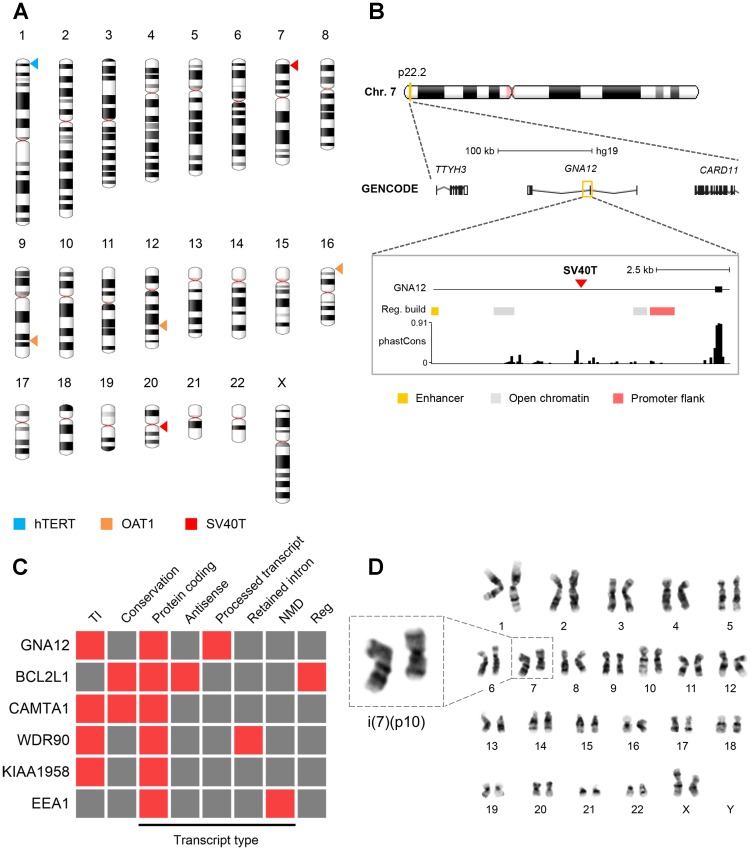
Viral integration sites and chromosomal stability. (**A**) Chromosomal distribution of the viral integration sites of the SV40T, hTERT and OAT1 transgenes. (**B**) Schematic representation of the integration of SV40T gene into *GNA12*. See Supplementary Figures 2–4 for schematic representation of remaining affected genes. Gene legend: untranslated region (empty box), exon (filled box), intron (line). (**C**) Functional consequence prediction of the viral integration sites. Presence and absence of a specific feature is shown in red and grey, respectively. Legend: TI = transcriptional interference, NMD = nonsense-mediated decay, reg = regulatory features. The phastCons P100 database was used to identify evolutionary conserved regions. (**D**) Cytogenetic analysis of ciPTEC-OAT1 at passage number 52. Representative female karyotype of a diploid cell showing an isochromosome for the short arm of chromosome 7.

Next, we evaluated the integration sites of the genes encoding for SV40T, hTERT and OAT1 and their consequences for ciPTEC-OAT1. For this, TLA was utilized to amplify the transgenes and their surrounding regions, from which precise integration sites were mapped (Figure 5A). We identified integrations sites located in the intronic regions of six endogenous genes. *SV40T* was integrated in *GNA12* (chromosome 7) (Figure 5B; Supplementary Figure 2A, 2C) and *BCL2L1* (chromosome 20) (Supplementary Figure 2B, 2C). The *hTERT* was stably integrated in the *CAMTA1* gene (chromosome 1) (Supplementary Figure 3), whereas *SLC22A6* (encoding OAT1) was integrated in *WDR90* (chromosome 16), *KIAA1958* (chromosome 9) and *EEA1* (chromosome 12) (Supplementary Figure 4). Several predictions were made regarding the functional consequences of these insertion sites (Figure 5C). Except for *BCL2L1* and *EEA1*, all endogenous genes are transcribed in the opposite direction compared to the inserted gene. A moderate degree of conservation was found for *BCL2L1* and *CAMTA1*, with phastCons 100-way scores of 0.149 and 0.273, respectively. The other sites were classified as not conserved. All integration sites were analysed with Ensembl Variant Effect Predictor. Only *BCL2L1* insertion site was found to be of relevance, containing both a regulatory-active site as well as being part of an antisense sequence (Figure 5C). The remaining transcript types were classified as low probability of being functionally relevant as they are either designated for degradation, such as nonsense-mediated decay (NMD), or the integrated sequence resides in a location where it would be spliced out.

For a final assessment of genomic abnormalities, ciPTEC-OAT1 at passage 52 were subjected to karyotype analysis. The cell population examined consisted of 68.2% near-tetraploid cells with the remainder being diploid. In-depth analysis of the diploid subpopulation (Figure 5D) shows the prevalence of an isochromosome abnormality concerning the p-arm of chromosome 7, i(7)(p10), which occurred in 2 out of 20 diploid metaphase spreads examined. Further, cells were re-examined at passage 62, where a complete shift towards near-tetraploidy was observed, with the presence of various complex aberrances among the entire cell population.

### Transgene integration does not affect endocytosis

**Figure 6:**
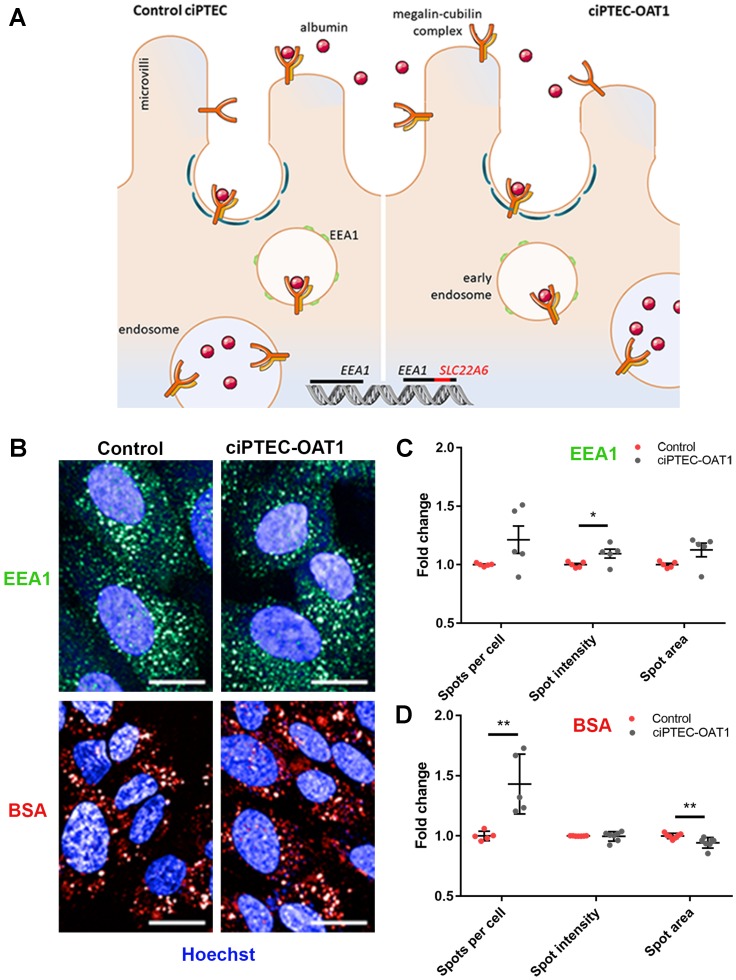
Endocytotic capacity of ciPTEC-OAT1. (**A**) Schematic representation of receptor-mediated endocytosis of albumin by control ciPTEC and ciPTEC-OAT1, showing the integration of OAT1-encoding gene (*SLC22A6*) within *EEA1*. (**B**) Representative immunofluorescence images of *EEA1* expression and endocytotic uptake of Alexa Fluor 647 labelled bovine serum albumin (BSA). Scale bars denote 20 μm. Quantification of the (**C**) *EEA1* expression and (**D**) BSA uptake by ciPTEC-OAT1 compared to control (parent ciPTEC). Values are normalized against control and expressed as the mean ± SEM of three independent experiments performed in triplicate. ^*^ *p* < 0.05, ^**^ *p* < 0.01 (unpaired two-tailed Student’s *t*-test).

One of the *SLC22A6* integration sites may have affected the function of one copy of *EEA1*, of which product, early endosome antigen (EEA1), is involved in endosomal trafficking (Figure 6A), a crucial function in PTEC [43]. However, endosomal clusters of EEA1, referred to as spots (Figure 6B–6C), did not differ between ciPTEC-OAT1 and the control parent cell line lacking the *EEA1*-affecting integration site. This maintained *EEA1* expression was accompanied by preserved endocytosis function, as assessed by the uptake of fluorescently labelled BSA in ciPTEC-OAT1 (Figure 6B). No differences between EEA1 expression levels and BSA spot intensity were found (Figure 6D).

### ciPTEC-OAT1 are not tumorigenic or oncogenic *in vivo*

**Figure 7:**
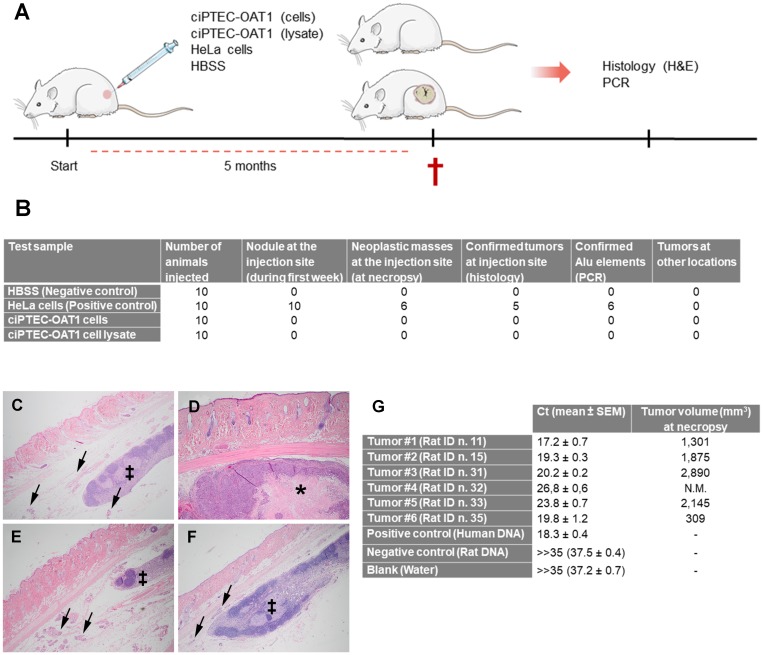
Tumorigenicity and oncogenicity of ciPTEC-OAT1. (**A**) Schematic representation of tumorigenicity and oncogenicity study *in vivo*. A total of 10^7^ cells (ciPTEC-OAT1 or HeLa) resuspended in 100 μl of HBSS were injected subcutaneously in the flank of the rats. In an additional group cell lysate derived from 10^7^ ciPTEC-OAT1 cells and resuspended in 100 μl of HBSS was injected per animal. In the negative control group rats received 100 μl of the vehicle (HBSS). Following the observational period of 5 months, animals were sacrificed (†) and histopathological and molecular (PCR) analyses were performed to confirm tumor formation and origin, respectively. (**B**) Summary of ciPTEC-OAT1 tumorigenicity and oncogenicity study results. (**C–F**) Representative pictures of histopathological analysis of the injection sites, performed by eosin and hematoxylin staining. (**C**) Negative control group injection site showing normal skin with subcutaneous lymph node (‡) and mammary tissue (arrows). (**D**) Positive control group injected with HeLa cells, showing the presence of anaplastic carcinoma with central area of necrosis (^*^) expanding in subcutaneous tissue at the site of injection. (**E**) Experimental tumorigenicity group injected with ciPTEC-OAT1 cells, presenting normal skin with subcutaneous lymph node (‡) and mammary tissue (arrows). (**F**) Oncogenicity group injected with ciPTEC-OAT1 cell lysate, showing normal skin with subcutaneous lymph node (‡) and mammary tissue (arrows). Pictures taken at magnification 2×. (**G**) PCR analysis of human-specific *Alu* elements confirming human origin of identified tumors and tumor volume (mm^3^) at necropsy. Ct values of all tumor samples were similar to that obtained for positive control human genomic DNA. Both the negative control (rat genomic DNA) and blank sample produced Ct values higher than 35. Limit of the blank [74], defined as the highest signal expected to be found when a blank sample containing no human DNA is tested, had a Ct value of 37.2 ± 0.71. N.M. (not measurable).

Finally, the tumorigenic and/or oncogenic potential ciPTEC-OAT1 were studied *in vivo* using nude athymic rats, and performed according to the World Health Organization (WHO) guidelines [44]. Tumorigenicity was evaluated by subcutaneous injection of 10^7^ living cells, and oncogenicity was assessed by injection of cell lysates originating from the same number of cells (Figure 7A). HeLa cells produced palpable but not measurable nodules within the first week of injection in all 10 rats. Over time, in 6 out of 10 animals the nodules progressed into larger neoplastic masses that, by histological analysis, were confirmed to be anaplastic carcinomas in 5 out of 6 cases (Figure 7B, 7D). In the remaining animal, we could not histologically confirm carcinoma formation, most likely attributable to a small-sized, non-measurable neoplastic mass at necropsy. PCR analysis confirmed the presence of human specific *Alu* elements in all identified carcinomas, and also in the rat without histological evidence of carcinoma (Figure 7G). Importantly, the subcutaneous injection of HBSS (vehicle), ciPTEC-OAT1 or the cell lysate did not lead to nodule formation (Figure 7B–7F) and did not compromise animal well-being (Supplementary Figure 5). Analysis showed no histological lesion in the skin, adnexa, subcutaneous tissue and lymph nodes at the site of injection, nor any neoplastic formations in other major organs including liver, lungs, colon, spleen, mesenteric lymph nodes, and kidneys. This suggests that rats were not susceptible to spontaneous tumour formation and that ciPTEC-OAT1, in the given animal model, do not exert tumorigenic or oncogenic potential.

## DISCUSSION

In the present study, we mechanistically and functionally confirmed that ciPTEC-OAT1 behave in accordance with conditional immortalization. After culture at non-permissive temperature they lose proliferative capacity and show contact inhibition. We found no indication for an important effect of transgene genomic integrations on endogenous gene expression or function. Although chromosomal aberrations could be demonstrated after multiple cell culture passages, there were no signs of *in vivo* tumorigenicity or oncogenicity.

The presence of residual SV40T, even after 7 days of culture at the non-permissive temperature, is in line with the known thermolabile characteristics of the SV40T mutant, as it will only be completely inactivated above 39.5° C [18]. Furthermore, considering BAK application and in case of a potential temperature drop during hemodialysis treatment, we observed a slight, but not significant, rebound in SV40T expression after re-exposing fully matured cells for 4 h at 33° C, however, this has no apparent effect on cell proliferation. Moreover, we showed that cell proliferation is dependent on SV40T expression as cells maintained at permissive temperature were highly proliferative compared to cells incubated at non-permissive temperature. This is in line with the mechanism of action of SV40T, which is known to interfere with Rb and p53 pathways thus driving cell proliferation [20]. Finally, the susceptibility of cells to undergo p53-mediated apoptosis at non-permissive temperature was confirmed using nutlin-3a, a compound that selectively induces p53 by inhibiting its degradation via Mdm2 [45, 46], suggesting normal p53 activity and apoptosis regulation at non-permissive temperature.

We examined the presence of contact inhibition in ciPTEC-OAT1 for two reasons. Firstly, non-transformed epithelial cells are expected to be responsive to growth-regulatory signals and undergo contact inhibition. Failure to display this would reflect cancerous cell behaviour and thus raise safety concerns [42]. Secondly, eyeing the potential use of ciPTEC-OAT1 in a BAK device [27], overgrowth of the cell monolayer could result in clogging of the tubules of the BAK device or affecting the epithelial barrier function. Our results showed that ciPTEC-OAT1 undergo contact inhibition and do not grow beyond the expected monolayer, even when they are cultured for long periods of time at permissive temperature. The observed absence of multi-layered growth of ciPTEC-OAT1 could also be explained by the presence of a crowding-induced live cell extrusion mechanism that helps maintaining homeostatic cell numbers in the epithelium [47].

Employing a soft-agar assay, known to correlate closely to *in vivo* tumour-forming ability of cells [48], we demonstrated that ciPTEC-OAT1 do not proliferate in an anchorage-independent manner. Small colonies observed sporadically were not progressively growing and exceeding 35 μm in diameter, indicating small cell clumps. In addition, we observed that the number of single cells present in the agarose at the beginning of the experiment decreased over the 4 weeks period of culturing, suggesting that some cells were dying, possibly due to anoikis [49]. However, this observation will be confirmed in future studies. In contrast to ciPTEC-OAT1, HeLa cells grew in absence of anchorage as described for cancer cell types [42].

Many assays have been described to study metastatic behaviour of malignant cells *in vitro* [50]. A requirement for metastasis is that cells are capable of invasion, *i.e.* migration through an ECM barrier in which movement is primarily limited to the cell’s ability to proteolytically degrade its surroundings, though amoeboid motility has also been reported depending on the (micro-)environment [51, 52]. Here, single ciPTEC-OAT1 cells were confined to a 3D environment that consisted of growth factor-reduced Matrigel™, using FCS-containing complete medium as a chemoattractant. The majority of cells lacked signs of invasive behaviour, though in a small subset we observed mesenchymal cell-like movement during the 24 h incubation. But studies have shown that this type of movement also plays an important role in tissue repair [53]. Furthermore, it should be emphasized that metastasis is a complex multi-step process, involving detachment from the cell bulk, intravasation into the systemic circulation, survival in a relatively harsh environment (e.g. lacking anchorage and presence of immune surveillance) and finally, extravasation into a distant tissue or organ [52]. No single *in vitro* assay fully recapitulates the complete chain of these events [50]. However, our *in vivo* results further support an absence of tumorigenic and metastatic potential of the cells.

The transgenes integration mediated by retroviral transduction could potentially lead to cell transformation and oncogenesis [37, 54]. Understanding functional consequences of the viral integration of the SV40T gene, *hTERT* and S*LC22A6* transgenes is far from trivial. In contrast to protein-coding sequences, the function of non-coding DNA remains largely unknown and the annotation of regulatory elements is often based on predictive models. Ensembl’s Regulatory Build is a good example, taking into account epigenetic markers, transcription-factor binding sites and DNase I hypersensitive sites (DHS), amongst other features, to define regulatory regions [55]. The limitations become clear when considering the integration site of *BCL2L1*, a gene involved in both pro- and anti-apoptotic signaling through its two protein isoforms, Bcl-xS and Bcl-xL [56]. The Regulatory Build categorizes this area as a promoter-flanking region, despite its location being approximately 27 kb downstream from the actual promoter. The activity of regulatory elements tends to be cell-type specific, which makes the impact estimation through an *in-silico* approach particularly challenging. Our results demonstrating that ciPTEC-OAT1 remain subjected to the intrinsic apoptosis pathway, of which Bcl2l1 is a key regulator [56], suggest that the viral insertion did not reduce the cell’s capacity to undergo apoptosis. Moreover, four out of six transgenes are integrated in the opposite DNA strand. A difference in orientation can cause transcription machineries to converge and collide, a process termed transcriptional interference (TI). This generally manifests itself in decreased transcript levels [57, 58]. However, this type of integration also has a protective effect as it allows the transgene to be spliced out, resulting in an intact messenger RNA (mRNA) of the endogenous gene [57]. On the other hand, the transgenes of which the orientation matches that of the endogenous gene, in case of *BCL2L1* and *EEA1*, could potentially lead to premature halt of endogenous genes transcription due to the presence of a termination signal in the long-terminal repeats (LTRs) of the viral vectors, leading to a truncated transcript of the endogenous gene [38]. However, it should be noted that one healthy allele remained for each affected gene, possibly limiting the impact of integration. In support, we demonstrated an intact endocytotic capacity in ciPTEC-OAT1 proving the unaltered expression and function of *EEA1*.

Karyotyping of ciPTEC-OAT1 at passages 52 and 62 showed the presence of a growing subpopulation of near-tetraploid cells with various complex chromosomal aberrations. Through its interaction with Bub-1, a spindle assembly checkpoint protein, SV40T can breach genomic integrity and induce tetraploidy [59]. Although this interaction appears to be unnecessary for immortalization, it has been demonstrated to trigger oncogenic transformation [60]. While the latter seemed absent in ciPTEC-OAT1, chromosomal abnormalities could have been avoided [18]. Still, SV40T as a tool for immortalization requires additional scrutiny, warranting a case-by-case evaluation of its impact on the chromosomal stability. This is especially important from a clinical perspective, as chromosomal instability could affect safety characteristics of a cell line. The creation of a SV40T mutant (U19dl89-97tsA58) that lacks the interaction site with Bub-1 is a promising development [61].

WHO guidelines regarding cell-based therapies suggest that all cell types intended for therapeutic purposes should be genetically stable as otherwise they would impose a significant risk regarding cell function and tumorigenic potential [44]. It should be noted that ciPTEC-OAT1 have previously undergone a rigorous functional assessment, showing that, at least from the functional perspective, cells remain stable over a wide range of passages [26]. Despite chromosomal aberrations, these cells differentiate in mature cell monolayers exhibiting mulitple PTEC-related functions, including epithelial barrier formation, protein uptake, vitamin D activation, and transport of uremic metabolites [26, 27, 62].

Further, in accordance to the WHO regulations [44], we addressed tumorigenic and oncogenic potential of ciPTEC-OAT1 *in vivo*. During the 5 months follow-up, our negative control group (vehicle control) confirmed the absence of spontaneous tumour formation. The guidelines also suggest that 90% of the animals within the positive control group should develop progressively growing tumours at the injection site. In all animals of the positive control group nodules appeared within the first week after HeLa cell injections, but only 50% of animals developed histologically and 60% PCR-confirmed HeLa derived tumours, indicating susceptibility of the animal model to grow tumour xenografts. Moreover, according to the guidelines, at least 20% of the animals within the test group should develop tumours in order to consider a particular cell type to be tumorigenic or oncogenic. Given that none of the animals developed tumours in the two test groups, we carefully conclude that in the athymic nude rat model, ciPTEC-OAT1 did not possess tumorigenic or oncogenic potential confirming our *in vitro* results.

Finally, considering that possible clinical use of these cells would only be in a context of an extracorporeal medical device and not direct transplantation, the altered karyotype and rare events of invasion observed *in vitro*, provided proper cell function, should not pose an extreme safety threat.

In conclusion, by showing that ciPTEC-OAT1 do not portray fundamental characteristics of oncogenically transformed cells, do not present negative consequences of viral transductions and genomic transgene integrations, such as insertional mutagenesis, nor possess tumorigenic capacity *in vivo*, the present study lays an important foundation towards validating the safety of a conditionally immortalized cell line for clinical application as cell-based renal replacement therapy.

## MATERIALS AND METHODS

### Materials

All reagents were obtained from Sigma-Aldrich (Zwijndrecht, the Netherlands) unless stated otherwise.

### Cell culture

Parent cell line (ciPTEC) and its OAT1-overexpressing derivate (ciPTEC-OAT1) were maintained in culture as described previously [26]. Culture of HeLa cells (ECACC, cat. nr. 93021013) is described in Supplementary Detailed Methods.

### Western blot analysis of SV40T

Protein expression of SV40T was analysed by Western blotting as described [16] and in Supplementary Detailed Methods.

### Cell cycle analysis

Cells fixed in ice-cold 70% (v/v) ethanol were stained with 40 μg/mL propidium iodide (PI) solution for 30 min and DNA content of >10 000 cells per condition was measured using FACSCanto II flow cytometer (BD Biosciences, San Jose, CA, USA), as described in Supplementary Detailed Methods.

### Apoptosis evaluation

Cell viability and caspase-3/7 expression, as indicators of apoptosis, were determined using PrestoBlue^®^ cell viability (Life Technologies, Paisly, UK) and CellEvent™ Caspase-3/7 Green detection (Invitrogen, Eugene, OR, USA) reagents, respectively, following manufacturer’s instructions. Details are reported in Supplementary Detailed Methods.

### Contact inhibition

Contact inhibition and multi-layered cell growth was determined by performing a z-stack imaging of Hoechst 33342 (1 μM) labelled cells by means of Cell Voyager 7000 (CV7000) confocal microscope (Yokogawa Electric Corporation, Tokyo, Japan). An ImageJ plugin was developed to quantify the surface area covered by cell multi-layers (Supplementary Method 1), as described in Supplementary Detailed Methods.

### CiPTEC-OAT1 culture on hollow fiber membranes

Cell proliferation in a 3D environment was assessed by culturing cells for 28 days on L-DOPA (2 mg/ml) and collagen IV (25 μg/ml) double-coated microPES hollow fiber membranes (HFM; Membrana GmbH, Wuppertal, Germany), as described previously [27, 63, 64] and in Supplementary Detailed Methods.

### Soft agar assay

Colony-forming ability of cells in anchorage-independent conditions was assayed in a similar manner as described by Borowicz *et al.* [41]. Procedure is described in Supplementary Detailed Methods.

### Single cell invasion assay

The invasion assay was based on the protocol described by Zaman *et al.* [51]. Cell tracking analysis was performed using Fiji’s TrackMate plugin to determine the speed of motion across acquisitions, as well as the average speed throughout the experiment [65, 66]. Cells having a speed of > 6 μm/h were classified as invasive. We manually validated this threshold for optimum discrimination between invasive and non-invasive cells. Inclusion and exclusion criteria were applied as previously described [51]. All data were processed in MySQL 5.6.17 (Oracle, Redwood City, CA, USA). Procedure is described in Supplementary Detailed Methods.

### Targeted locus amplification for viral integration sites

The ciPTEC-OAT1 cell line was stably transduced using three viral vectors [16, 26], warranting an investigation into the occurrence of cell behavior-altering insertional mutagenesis. Determination of the exact location of the integrated transgenes was performed by Cergentis B.V. (Utrecht, the Netherlands) using targeted locus-amplification (TLA) technology as described [67]. Data was analysed using Ensembl’s genome browser in conjunction with the regulatory build [55, 68]. To predict the functional consequences of the integration sites, the Ensembl Variant Effect Predictor was utilized [69].

### Cytogenetic analysis

Metaphase spreads of ciPTEC-OAT1 were G-banded and analysed for abnormalities (Cell Guidance Systems, Cambridge, UK). Approximately 20 metaphase spreads were analysed per experiment. Sample preparation is described in Supplementary Detailed Methods.

### Endocytosis

Endocytosis was evaluated by evaluating early endosome antigen (EEA1) expression and bovine serum albumin (BSA) uptake, similarly to [70] and reported in Supplementary Detailed Methods.

### Tumorigenicity and oncogenicity evaluation *in vivo*

Animal procedures were approved by the Ethics Committee of Animal Research of Utrecht University, Utrecht, The Netherlands (CCD approval number AVD108002017879). Male (*n* = 20; 4 weeks old, weighing between 103 and 172 g) and female (*n* = 20; 4 weeks old, weighing between 94 and 133 g) athymic nude rats (Hsd:RH-Foxn1^rnu^; Envigo, Horst, Netherlands) were maintained in the Central Laboratory Animal Research Facility (GDL, Utrecht, Netherlands), and housed in individually ventilated cage units at RT under a 12 h light/dark cycle. Food and water were provided *ad libitum*. All animals were treated according to IVD and CCD guidelines and all efforts were made to minimize suffering. Animals were euthanized by pentobarbital (Faculty of Veterinary Medicine, Utrecht, the Netherlands) overdose via intraperitoneal injection, followed by cervical dislocation as soon as animals became unconscious. Details are reported in Supplementary Detailed Methods.

### Histopathological analysis

First, formalin-fixed tissues and organs were examined macroscopically for presence of abnormalities. Afterwards, formalin-fixed tissues were subjected to further microscopical histological analysis as described [71, 72]. Examination was performed by board-certified veterinary pathologists of the Dutch Molecular Pathology Centre (Department of Pathobiology, Faculty of Veterinary Medicine, Utrecht, the Netherlands). Representative images were taken using Olympus BX45 microscope equipped with DP25 camera (Leiderdorp, the Netherlands) with 2× magnification.

### DNA extraction and PCR analysis

Human origin of observed tumors was confirmed by detection of human specific *Alu* elements [73], using Real-Time PCR, following manufacturer’s instructions. Details regarding sample preparation, reaction protocol and primers used are described in Supplementary Detailed Methods.

### Statistical analysis

Statistical analysis was performed using GraphPad Prism 7.0 (GraphPad Software, Inc., La Jolla, CA, USA), unless stated otherwise. Data are presented as mean ± standard error of the mean (SEM) of three independent experiments performed in triplicate, unless stated otherwise. Significance was evaluated using the unpaired two-tailed Student’s *t*-test or one-way ANOVA followed by Dunnett’s multiple comparison test where appropriate. *P*-values < 0.05 were considered as significant. Where appropriate, significance is denoted as ^*^(*p* < 0.05), ^**^(*p* < 0.01) and ^***^(*p* < 0.001).

## SUPPLEMENTARY MATERIALS



## Abbreviations

BAK: bioartificial kidney
BCL2L1: BCL2 Like 1
BSA: bovine serum albumin
ciPTEC: conditionally immortalized proximal tubule epithelial cells
ECM: extracellular matrix
EEA1: early endosome antigen
ESKD: end-stage kidney disease
HFM: hollow fiber membranes
hTERT: human telomerase
L-DOPA: levodopa
LLC-PK1: Lilly Laboratories cell porcine kidney 1
LTR: long-terminal repeats
MDCK: Madin-Darby canine kidney
OAT1: organic anion transporter 1
PTEC: proximal tubule epithelial cells
SV40T: SV40 large T antigen
TLA: targeted locus amplification
