# Supplementary material for: Safety evaluation of conditionally immortalized cells for renal replacement therapy
Source: Oncotarget. 2019 Sep 3;10(51):5332–48. doi: 10.18632/oncotarget.27152 (PMC6731099; doi:10.18632/oncotarget.27152)
Supplement: Supplementary file 1 [file oncotarget-10-5332-s001.pdf]

Safety evaluation of conditionally immortalized cells for renal replacement therapy

SUPPLEMENTARY MATERIALS

Supplementary Detailed Methods

Cell culture

Both the parent cell line (ciPTEC) and its OAT1-overexpressing derivate (ciPTEC-OAT1) were cultured in Dulbecco's Modified Eagle Medium/Nutrient Mixture F-12 (1:1 DMEM/F-12) (Gibco, Life Technologies, Paisley, UK) supplemented with 10% fetal calf serum (FCS) (Greiner Bio-One, Alphen aan den Rijn, The Netherlands), 5 μg/mL insulin, 5 μg/mL transferrin, 5 μg/mL selenium, 35 ng/mL hydrocortisone, 10 ng/mL epidermal growth factor and 40 pg/mL tri-iodothyronine, creating complete culture medium, without addition of antibiotics. The cells were seeded at a density of 48 000 and 55 000 cells/cm2, respectively. Seeded cells were kept at 33° C for 1 day prior to 7 days of maturation at 37° C. HeLa cells (ECACC, cat. nr. 93021013) were propagated at 37° C using high glucose Dulbecco's Modified Eagle Medium (DMEM; Gibco Life Technologies, Paisly, UK), supplemented with 10% (v/v) FCS and 1% (v/v) 5 000 U/mL penicillin/streptomycin, and seeded at a density of 55 000 cells/cm^2. All cell lines were passaged using Accutase® solution and incubated in a humidified atmosphere containing 5% (v/v) CO_2.

Western blot analysis of SV40T

After seeding, ciPTEC-OAT1 were kept at 33° C or matured for either 1 or 7 days at 37° C. In addition to this, fully matured cells were also incubated for additional 4 h at 33° C to simulate the effects of a transient temperature drop during hemodialysis. Following removal of culture medium and two washing steps in cold PBS, cell lysate was obtained through 5 min incubation on ice in 350 μl radioimmunoprecipitation assay (RIPA) lysis buffer (Thermo Scientific, Waltham, MA, USA), containing 1% (v/v) protease and phosphatase inhibitors (Halt Protease Inhibitor Cocktail, Halt Phosphatase Inhibitor Cocktail; Thermo Scientific, Waltham, MA, USA). Cell lysates were collected with a cell scraper (Costar 3010; Corning, NY, USA) and centrifuged at 14 000 × g for 15 min at 4° C. The supernatant's protein content was, subsequently, determined using the Pierce™ BCA Protein Assay Kit (Thermo Scientific, Waltham, MA, USA) and samples were stored at −80° C until further usage. Prior to Western blot analysis, cell lysates were denatured and reduced in 2% β-mercaptoethanol for 5 min at 95° C using a T100 thermocycler (Bio-Rad, Veenendaal, the Netherlands). A total 15 μg of proteins per sample were separated by 4–20% sodium dodecyl sulfate polyacrylamide gel electrophoresis (SDS-PAGE; Bio-Rad, Veenendaal, the Netherlands) at 200 V and wet-transferred to a 0.2 μm pore-sized polyvinylidene difluoride (PVDF) membrane (Bio-Rad, Veenendaal, the Netherlands) for 1 h at 100 V. The membrane was subsequently blocked for 1 h with 5% fat-free dry milk (Nutricia, Zoetermeer, the Netherlands) in PBS/0.1% Tween-20 (PBS-T; Millipore, Etten-Leur, the Netherlands). To allow protein quantification, the membrane was probed with mouse anti-SV40T (1:2 000; Santa Cruz Biotechnology, Dallas, TX, USA) and rabbit anti-GAPDH (1:5 000) primary antibodies, followed by HRP-conjugated secondary antibodies for SV40T (1:5 000, rabbit anti-mouse; Dako, Carpinteria, CA, USA) and GAPDH (1:5 000, goat anti-rabbit; Dako, Carpinteria, CA, USA). Finally, chemiluminescence was developed with Clarity Western ECL Substrate (Bio-Rad, Veenendaal, the Netherlands), images acquired using ChemiDoc™ MP Imaging System (Bio-Rad Laboratories) and data analyzed by means of Image Lab software (version 5.2, Bio-Rad Laboratories). SV40T expression was quantified using Fiji software through densitometric analysis of imaged bands [1], using GAPDH for normalization. Data were presented as relative expression, using as a reference cells grown at permissive temperature.

Cell cycle analysis

The effect of SV40T on the proliferation of ciPTEC-OAT1 was studied in a subconfluent and confluent state. To this end, cells were seeded at a low (10 000 cells/cm^2) and regular density and maintained in culture for various durations. Next, the harvested cells were fixed in ice-cold 70% (v/v) ethanol, washed twice with PBS, and incubated in a solution consisting of 40 μg/mL propidium iodide (PI), 0.1% (v/v) Triton X-100 and 100 μg/mL RNAse A for 30 min. DNA content of at least 10 000 cells per condition was measured on a FACSCanto II flow cytometer (BD Biosciences, San Jose, CA, USA) and the cell cycle distribution was

analyzed with FlowLogic 6.0 (Inivai Technologies, Melbourne, Australia).

Apoptosis evaluation

CiPTEC-OAT1 at permissive (33° C) and non-permissive (37 °C) temperatures were exposed to nutlin-3a (Axon Medchem, Groningen, the Netherlands) to induce p53-mediated apoptosis [2, 3]. Nutlin-3a was diluted from a 10 mM dimethyl sulfoxide (DMSO)-dissolved stock to desired concentrations. Following 24 h exposure, cell viability and caspase-3/7, as indicators of apoptosis, were determined using PrestoBlue® cell viability (Life Technologies, Paisly, UK) and CellEvent™ Caspase-3/7 Green detection (Invitrogen, Eugene, OR, USA) reagents respectively. For cell viability evaluation, cells were rinsed with HBSS and incubated with PrestoBlue® cell viability reagent or CellEvent™ Caspase-3/7 Green detection reagent at a concentration of 8 µM (diluted in HBSS containing 5% FCS) for 1 h in the dark. Afterwards, the fluorescence was measured using a fluorescent microplate reader (Fluoroskan Ascent FL, Thermo Fisher Scientific, Vantaa, Finland) at excitation/emission wavelengths of 530/590 nm for cell viability and 492/518 nm for caspase-3/7 detection. The obtained values were corrected for background (absence of cells), normalized to untreated cells and presented as relative cell viability and caspase-3/7 detection.

Contact inhibition

To investigate contact inhibition, ciPTEC-OAT1 and HeLa cells were cultured in 96-well plates (Costar 3599; Corning, NY, USA) at 33° C and 37° C for 4 weeks. The cells were, subsequently, fixed with 4% (w/v) PFA for 15 min and nuclei were stained with 1 µM Hoechst 33342 for 15 min. All aforementioned compounds were dissolved in phosphate-buffered saline (PBS; Lonza, Verviers, Belgium). A z-stack, spanning 100 µm with a 1 µm slice interval, was acquired on a Cell Voyager 7000 (CV7000) confocal microscope (Yokogawa Electric Corporation, Tokyo, Japan) with 10× magnification. At least 10 fields per condition were analyzed using ImageJ software 1.40 g. An ImageJ plugin was developed to quantify the surface area covered by cell multi-layers (Supplementary Method 1), which signatures excessive proliferation and loss of contact inhibition. Values were expressed as percentage of total analyzed surface.

CiPTEC-OAT1 culture on hollow fiber membranes

To investigate cell proliferation in a 3D environment relevant to BAK, ciPTEC-OAT1 were cultured on microPES hollow fiber membranes (HFM; MicroPES type TF10 hollow fiber capillary membranes with wall thickness 100 µm, inner diameter 300 µm, max pore size 0.5 µm; Membrana GmbH, Wuppertal, Germany). Following L-DOPA (2 mg/ml) and collagen IV (25 µg/ml) double-coating strategy [4, 5], HFM were seeded with cells as described previously [6]. After 28 days culture at either permissive or non-permissive temperature HFM containing cells were fixed with 4% (w/v) paraformaldehyde (PFA; VWR International, Amsterdam, the Netherlands) dissolved in PHEM buffer (120 mM PIPES, 50 mM HEPES, 4 mM $MgCl_2$, 20 mM EGTA) for 15 min and then washed in Hank's Balanced Salt Solution (HBSS; Gibco Life Technologies, Paisly, UK). Next, ProLong™ Gold antifade reagent containing DAPI (Life Technologies, Eugene, OR, USA) was used for nuclear staining, and to mount the fibers on the Willco glass bottom dishes (WillCo Wells B.V., Amsterdam, The Netherlands). Finally, cells were imaged using confocal microscope (Leica TCS SP8 X, Leica Microsystems CMS GmbH, Wetzlar, Germany) and analyzed by means of Leica Application Suite X software (Leica Microsystems CMS GmbH).

Soft agar assay

Colony-forming ability of cells in anchorage-independent conditions was assayed in a similar manner as previously described by Borowicz *et al.* [7]. Briefly, a 6-well plate (Costar 3506; Corning, NY, USA) was coated with 1.5 ml 0.5% (w/v) agarose that was diluted from an autoclaved 2% (w/v) agarose stock in cell culture medium. Upon solidification for 30 min at room temperature (RT), 10 000 ciPTEC-OAT1 or HeLa cells were mixed with the agarose stock solution to a final volume and concentration of 1.5 ml and 0.3% (w/v), respectively, and layered on top of the coating. After solidification, agarose layers containing cells were covered with 1 ml of complete culture medium and incubated for 4 weeks at either 33 °C or 37 °C. Colonies were manually counted in at least 15 fields per condition, using an Axiovert 25 bright-field microscope (Carl Zeiss, Oberkochen, Germany) at 10× magnification. Representative images of colonies were taken on CV7000 confocal microscope by phase-contrast microscopy equipped with a 20× objective.

Single cell invasion assay

The invasion assay was based on the protocol described by Zaman *et al.* [8]. Matured ciPTEC-OAT1 were harvested, washed twice in HBSS and resuspended in serum-free medium (SFM). 125 µl of cell suspension, containing 40 000 cells in SFM, was thoroughly mixed with an equal volume of growth factor reduced Matrigel™ (Corning, NY, USA) and transferred to a 24-well plate (Costar 2524; Corning, NY, USA). Special care was taken to prevent the formation of air bubbles, which could hinder imaging and affect cell behaviour [8, 9], hence the final

gel thickness was approximately 200 μm. Following 1 h incubation at 37° C, the solidified gel was gently covered with 250 μl of complete culture medium, serving as a chemoattractant. Time-lapse imaging was performed using CV7000 confocal microscope under environmentally controlled conditions (humidified, 5% (v/v) CO_2 and 37° C), by taking pictures at 30 min intervals for 24 h. Each acquisition entailed a phase-contrast z-stack across the entire height of the gel, captured at 20× magnification with a slice thickness of 5 μm. Cell tracking analysis was performed using Fiji's TrackMate plugin to determine the speed of motion across acquisitions, as well as the average speed throughout the experiment [1, 10]. Cells having a speed of > 6 μm/h were classified as invasive. We manually validated this threshold for optimum discrimination between invasive and non-invasive cells. Inclusion and exclusion criteria were applied as previously described [8]. All data were processed in MySQL 5.6.17 (Oracle, Redwood City, CA, USA).

Cytogenetic analysis

Metaphase spreads of ciPTEC-OAT1 were G-banded and analyzed for abnormalities (Cell Guidance Systems, Cambridge, UK). For sample preparation, log-phase cells (approximately at 70% confluence) were growth-arrested by treatment with 0.1 μg/ml colcemid (KaryoMAX, Gibco Life Technologies, Paisly, UK) at 37° C for 15 h. Next, the harvested cells were treated with a hypotonic solution consisting of 75 mM KCl in HBSS. This was followed by fixation in 3:1 (v/v) methanol/acetic acid solution. To assess chromosomal stability, the experiment was performed twice over a period spanning 10 cell culture passages. Approximately 20 metaphase spreads were analyzed per experiment.

Endocytosis

To study endocytosis, bovine serum albumin (BSA) uptake and early endosome antigen (EEA1) were evaluated. First, ciPTEC-OAT1 and ciPTEC cells were seeded in black special optics 96-well plates (Corning, NY, USA) and exposed to 5 μg/ml BSA conjugated to Alexa Fluor 647 (Thermo Scientific, Waltham, MA, USA) for 1 h at 37° C. Nuclei were simultaneously stained with 1 μM Hoechst 33342. A 20 μm z-stack was acquired at 0.5 μm intervals using the CV7000 confocal microscope at 60× magnification (excitation at 405 nm and 640 nm, acquisition using 445/40 nm and 676/29 nm band-pass (BP) filters, respectively). Afterwards, the cells were fixed with 4% (w/v) PFA and permeabilized using 0.1% (v/v) Triton X-100. Following 30 min blocking in 1% (w/v) BSA at RT, the EEA1 was probed with a rabbit anti-human EEA1 primary antibody (1:200; Cell Signaling, Danvers, MA, USA) and donkey anti-rabbit secondary antibody conjugated with Alexa Fluor 488 (1:500; Invitrogen, Carlsbad, CA, USA). For imaging, the CV7000 microscope with the same z-stack settings, excitation laser 488 nm and acquisition channel 525/50 nm BP filter were used to image EEA1 expression in cells. Finally, uptake of the fluorescently labelled BSA and EEA1 expression were both quantified using Columbus™ Image Data Storage and Analysis software 2.7.1 (PerkinElmer, Groningen, the Netherlands) and expressed as number of spots per cell, average spot size and spot intensity.

Tumorigenicity and oncogenicity evaluation *in vivo*

Male (n = 20; 4 weeks old, weighing between 103 and 172 g) and female (n = 20; 4 weeks old, weighing between 94 and 133 g) athymic nude rats (Hsd:RH-Foxn1rnu; Envigo, Horst, Netherlands), maintained in the Central Laboratory Animal Research Facility (GDL, Utrecht, Netherlands), were used for *in vivo* tumorigenicity and oncogenicity studies. The rats were housed in individually ventilated cage units at RT under a 12 h light/dark cycle. Food and water were provided ad libitum. Animal procedures were approved by the Ethics Committee of Animal Research of Utrecht University, Utrecht, The Netherlands (CCD approval number AVD108002017879). All animals were treated according to IVD and CCD guidelines and all efforts were made to minimize suffering. All animals were euthanized by pentobarbital (Faculty of Veterinary Medicine, Utrecht, the Netherlands) overdose via intraperitoneal injection, followed by cervical dislocation as soon as animals became unconscious.

The rats were randomly divided into four treatment groups keeping the ratio of male and female rats 1:1 in each group. The first test group received ciPTEC-OAT1 cells for tumorigenicity evaluation, the second group of animals received ciPTEC-OAT1 cell lysates, which were obtained by repetitive freeze/thaw cycles, to study the oncogenic potential of ciPTEC-OAT1. For both tumorigenicity and oncogenicity groups, ciPTEC-OAT1 were used at passage 57. The remaining two groups served as a positive control group that received HeLa cells and a negative control group that was given HBSS to monitor for spontaneous tumor growth. A total number of 10^7 cells or lysate derived from the equivalent amount of cells was injected subcutaneously in the flank of the animals in a total volume of 100 μl of HBSS, using 1 ml syringes with a 25 G needle (Terumo Europe N.V., Leuven, Belgium). The rats in the negative control group were injected with 100 μl of HBSS. Injections were performed in random order and in blinded fashion for the operator. After injection, all rats were observed for a maximum of 23 weeks, during which the animals were palpated every two days to detect nodule formation and tumor development at the site of injection and to monitor for clinical symptoms (weight, anxiousness, paralysis and diarrhea). Moreover,

all animals were checked daily for their overall well-being. As soon as the tumors reached measurable size, the tumor length and width were measured using a calliper. Tumor volume was calculated using the formula: $1/2 \times \text{length (mm)} \times (\text{width (mm)})^2$. The rats were euthanized and necropsied when the humane endpoint (tumor mass of 4.2 cm in diameter or weight loss of more than 15% in two days) was reached, or when the tumors were of a sufficient size to consider them as progressively growing tumors. The site of injection, lungs, mesenteric lymph nodes, liver, spleen, kidneys and colon were harvested and examined by board-certified veterinary pathologists of the Dutch Molecular Pathology Centre (Department of Pathobiology, Faculty of Veterinary Medicine, Utrecht, the Netherlands). Immediately after harvesting, tissue and tumor samples were fixed in formalin or frozen in liquid nitrogen until further use.

Histopathological analysis

First, formalin-fixed tissues and organs were examined macroscopically for the presence of abnormalities. Afterwards, formalin-fixed tissues were paraffin-embedded and slices of approximately 5 µm thickness were stained with hematoxylin and eosin and subjected to further microscopical histological analysis as described previously [11]. Slides from all the aforementioned organs were analysed histologically for the presence of neoplastic lesions, as well as of any significant alterations [12]. Representative images were taken using Olympus BX45 microscope equipped with DP25 camera (Leiderdorp, the Netherlands) with 2× magnification. Histologically confirmed tumors were further characterized by PCR.

DNA extraction and PCR analysis

Human origin of observed tumors was confirmed by detection of human specific Alu elements, the short interspersed elements (SINEs) present in primate genomes [13] Q/, using Real-Time PCR. After being recovered from liquid nitrogen, tumor tissues were cut into small pieces weighing between 20 and 25 mg and used for DNA extraction. Following tissue homogenization in PBS, genomic DNA was extracted using QIAamp® DNA Mini kit (Qiagen GmbH, Hilden, Germany) according to manufacturer's instructions and quantified using the NanoDrop® ND-1000 spectrophotometer. Next, Real-time PCR was performed using 25 ng of each DNA sample. The reaction was carried out using the iQ SYBR® Green Supermix (Bio-Rad Laboratories, Hercules, CA, USA) as indicated in manufacturer's protocol and by means of CFX96™ Real-Time PCR Detection System (Bio-Rad Laboratories, Hercules, CA, USA). The reaction was performed by initial denaturation at 95° C for 10 min, 40 amplification cycles at 95° C for 15 sec, 62° C for 5 sec and 72° C for 15 sec, and followed by determination of melting curves by performing denaturation at 95° C for 30 sec, followed by complete annealing and a gradual increase in temperature starting from 60° C and reaching 95° C with a transition rate of 0.1° C/sec, as described previously [14]. The data were analyzed using Bio-Rad CFX Manager™ Software version 3.1 (Bio-Rad Laboratories, Hercules, CA, USA) and expressed as mean Ct values. Specific sense and anti-sense primers for AluYb8 element [15] Human (forward: CGAGGCGGGTGGATCATGAGGT; reverse: TCTGTCGCCCAGGCCGGACT) were synthesized by Biolegio (Nijmegen, The Netherlands). To avoid non-specific rodent DNA amplification, low primer concentration of 150 nM was used. In each PCR reaction a negative control, given by rat genomic DNA, and a blank sample containing water instead of DNA were included. This was used also to determine the limit of the blank which represents the highest signal obtained in samples containing no human DNA [16]. Primer specificity and linearity of the PCR assay were determined by 5-fold dilutions of the known human DNA sample.

REFERENCES

1. Schindelin J, Arganda-Carreras I, Frise E, Kaynig V, Longair M, Pietzsch T, Preibisch S, Rueden C, Saalfeld S, Schmid B, Tinevez JY, White DJ, Hartenstein V, et al. Fiji: an open-source platform for biological-image analysis. Nat Methods. 2012; 9:676–82. https://doi.org/10.1038/nmeth.2019. [PubMed]
2. Ohnstad HO, Paulsen EB, Noordhuis P, Berg M, Lothe RA, Vassilev LT, Myklebost O. MDM2 antagonist Nutlin-3a potentiates antitumour activity of cytotoxic drugs in sarcoma cell lines. BMC Cancer. 2011; 11:211:1–11. https://doi.org/10.1186/1471-2407-11-211. [PubMed]
3. Villalonga-Planells R, Coll-Mulet L, Martínez-Soler F, Castaño E, Acebes JJ, Giménez-Bonafé P, Gil J, Tortosa A. Activation of p53 by nutlin-3a induces apoptosis and cellular senescence in human glioblastoma multiforme. PLoS One. 2011; 6:e18588. https://doi.org/10.1371/journal.pone.0018588. [PubMed]
4. Ni M, Teo JC, Ibrahim MS, Zhang K, Tasnim F, Chow PY, Zink D, Ying JY. Characterization of membrane materials and membrane coatings for bioreactor units of bioartificial kidneys. Biomaterials. 2011; 32:1465–76. https://doi.org/10.1016/j.biomaterials.2010.10.061. [PubMed]
5. Oo ZY, Deng R, Hu M, Ni M, Kandasamy K, bin Ibrahim MS, Ying JY, Zink D. The performance of primary human renal cells in hollow fiber bioreactors for bioartificial kidneys. Biomaterials. 2011; 32:8806–15. https://doi.org/10.1016/j.biomaterials.2011.08.030. [PubMed]
6. Jansen J, Fedecostante M, Wilmer MJ, Peters JG, Kreuser UM, van den Broek PH, Mensink RA, Boltje TJ, Stamatialis D, Wetzels JF, van den Heuvel LP, Hoenderop

JG, Masereeuw R. Bioengineered kidney tubules efficiently excrete uremic toxins. Sci Rep. 2016; 6:26715. https://doi.org/10.1038/srep26715. [PubMed]

7. Borowicz S, Van Scoyk M, Avasarala S, Karuppusamy Rathinam MK, Tauler J, Bikkavilli RK, Winn RA. The soft agar colony formation assay. J Vis Exp. 2014; e51998. https://doi.org/10.3791/51998. [PubMed]

8. Zaman MH, Trapani LM, Sieminski AL, Mackellar D, Gong H, Kamm RD, Wells A, Lauffenburger DA, Matsudaira P. Migration of tumor cells in 3D matrices is governed by matrix stiffness along with cell-matrix adhesion and proteolysis. Proc Natl Acad Sci U S A. 2006; 103:10889–94. https://doi.org/10.1073/pnas.0604460103. [PubMed] Erratum in: Proc Natl Acad Sci U S A. 2006 Sep 12;103(37):13897. https://doi.org/10.1073/pnas.0606087103.

9. Buxboim A, Rajagopal K, Brown AE, Discher DE. How deeply cells feel: methods for thin gels. J Phys Condens Matter. 2010; 22:194116. https://doi.org/10.1088/0953-8984/22/19/194116. [PubMed]

10. Tinevez JY, Perry N, Schindelin J, Hoopes GM, Reynolds GD, Laplantine E, Bednarek SY, Shorte SL, Eliceiri KW. TrackMate: an open and extensible platform for single-particle tracking. Methods. 2017; 115:80–90. https://doi.org/10.1016/j.ymeth.2016.09.016. [PubMed]

11. Henriques U. Histological technique in routine histopathology. An opinion. Pathol Res Pract. 1981; 171:417–22. https://doi.org/10.1016/S0344-0338(81)80114-8. [PubMed]

12. McInnes EF, Scudamore CL. Review of approaches to the recording of background lesions in toxicologic pathology studies in rats. Toxicol Lett. 2014; 229:134–43. https://doi.org/10.1016/j.toxlet.2014.06.005. [PubMed]

13. Batzer MA, Deininger PL. Alu repeats and human genomic diversity. Nat Rev Genet. 2002; 3:370–79. https://doi.org/10.1038/nrg798. [PubMed]

14. Prigent J, Herrero A, Ambroise J, Smets F, Deblandre GA, Sokal EM. Human Progenitor Cell Quantification After Xenotransplantation in Rat and Mouse Models by a Sensitive qPCR Assay. Cell Transplant. 2015; 24:1639–52. https://doi.org/10.3727/096368914X681955. [PubMed]

15. Walker JA, Kilroy GE, Xing J, Shewale J, Sinha SK, Batzer MA. Human DNA quantitation using Alu element-based polymerase chain reaction. Anal Biochem. 2003; 315:122–28. https://doi.org/10.1016/S0003-2697(03)00081-2. [PubMed]

16. Armbruster DA, Pry T. Limit of blank, limit of detection and limit of quantitation. Clin Biochem Rev. 2008 (Suppl 1); 29:S49–52. [PubMed]

Supplementary Method 1: ImageJ plugin for the quantification of surface area containing multi-layered cell growth

```
// Step 1: Specify the name of the ImageJ window here

var windowName = "window";

selectWindow(windowName);

// Step 2: Convert to 8-bit

run("8-bit");

// Move up in the z-stack till single-layered cells are not visible

// Specify the slice (value depends on your experimental set-up)

setSlice(30);

selectWindow(windowName);

// Step 3: Enhance contrast

run("Brightness/Contrast...");

for (i = 0; i < 4; i++) {

      run("Enhance Contrast", "saturated=0.35");

}

run("Apply LUT", "stack");

selectWindow(windowName);

// Step 4: Blur the image to account for empty space between cells

// Remember, we're imaging nuclei, so inevitably there will be empty space (even
in a fully confluent plate)

run("Gaussian Blur...", "sigma=10 slice");

selectWindow(windowName);

// Step 5: Threshold the image to get pixels with cells (white) and pixels without
(black).

// The following code regarding thresholding is courtesy

// of Michael Schmid (Institut für Angewandte Physik, Technische Universität Wien)

percentage = 75;

nBins = 256;

resetMinAndMax();

getHistogram(values, counts, nBins);

nPixels = 0;
```

```
for (i = 0; i < counts.length; i++) {
  nPixels += counts[i];
}
nBelowThreshold = nPixels * percentage / 100;
sum = 0;
for (i = 0; i < counts.length; i++) {
  sum = sum + counts[i];
  if (sum >= nBelowThreshold) {
    setThreshold(values[0], values[i]);
    i = 99999999; // Break out of loop
  }
}
setOption("BlackBackground", false);
run("Convert to Mask", "method=Default background=Light only");
selectWindow(windowName);
getHistogram(values, counts, 256);
var results_c = nResults;
// Step 6: Output the results to the ImageJ console
setResult("Surface area containing multi-layered cell growth", results_c,
counts[255]);
setResult("Total surface area", results_c, counts[0] + counts[255]);
updateResults();
```

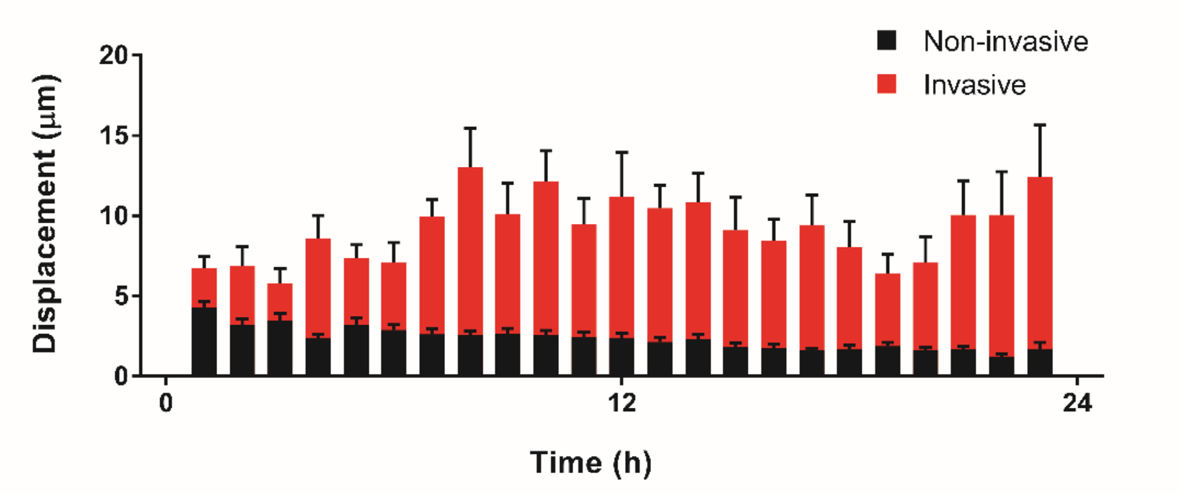

Supplementary Figure 1: Migration pattern of ciPTEC-OAT1. Cells migration and movement through Matrigel™ basement membrane matrix were monitored during 24 h time-lapse imaging. In graph, displacement (µm) of invasive and non-invasive cells at different time points during the 24 h imaging is shown.

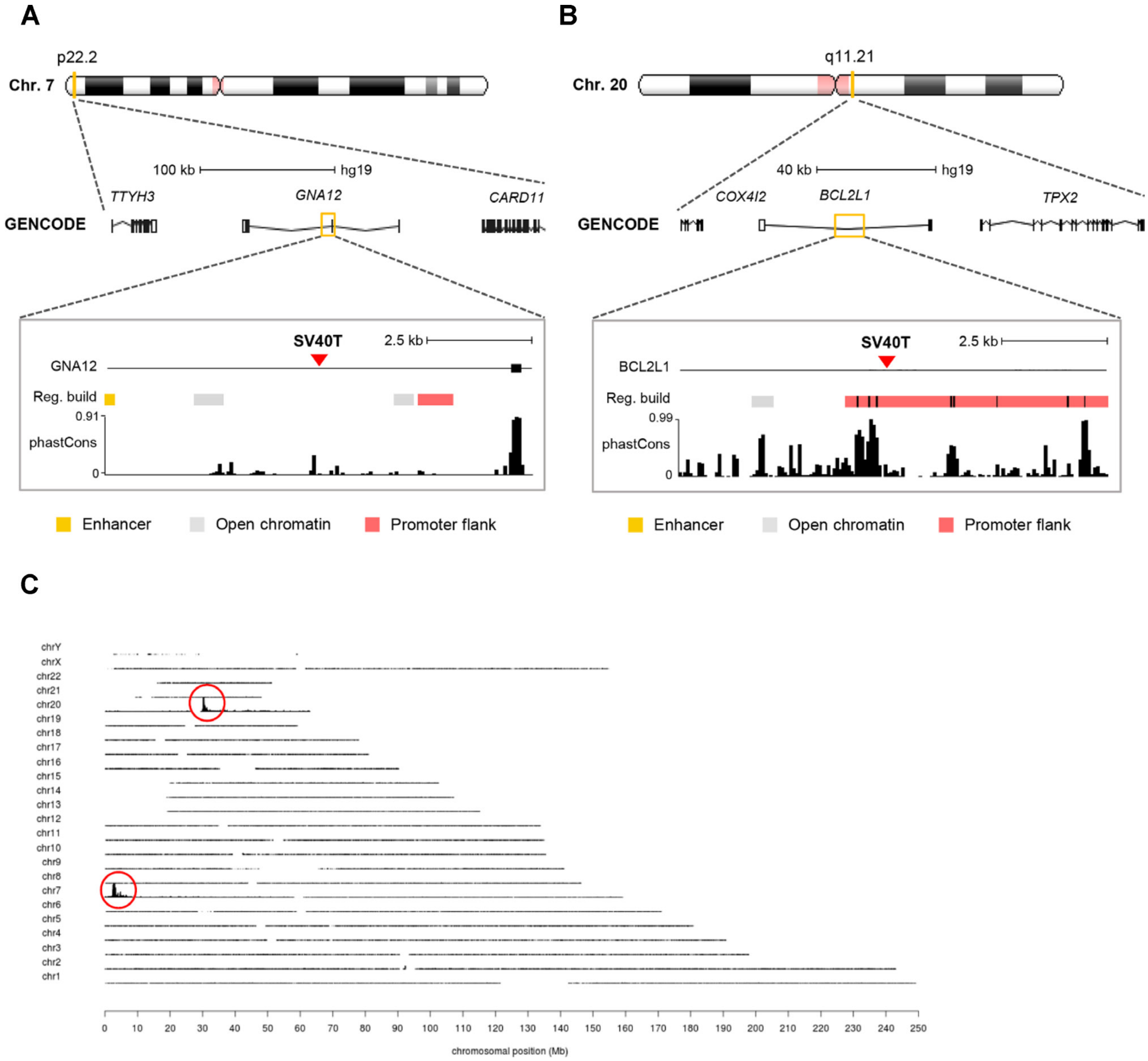

Supplementary Figure 2: Viral integration sites of theSV40T gene. The integration sites of the gene encoding SV40T within (**A**) *GNA12* and (**B**) *BCL2L1*. The phastCons P100 database was used to identify evolutionary conserved regions. Gene legend: untranslated region (empty box), exon (filled box), intron (line). (**C**) TLA sequence coverage across the human genome with different chromosomes indicated on the y-axis, and the chromosomal position on the x-axis. The position of the integration sites of the SV40T gene are encircled in red.

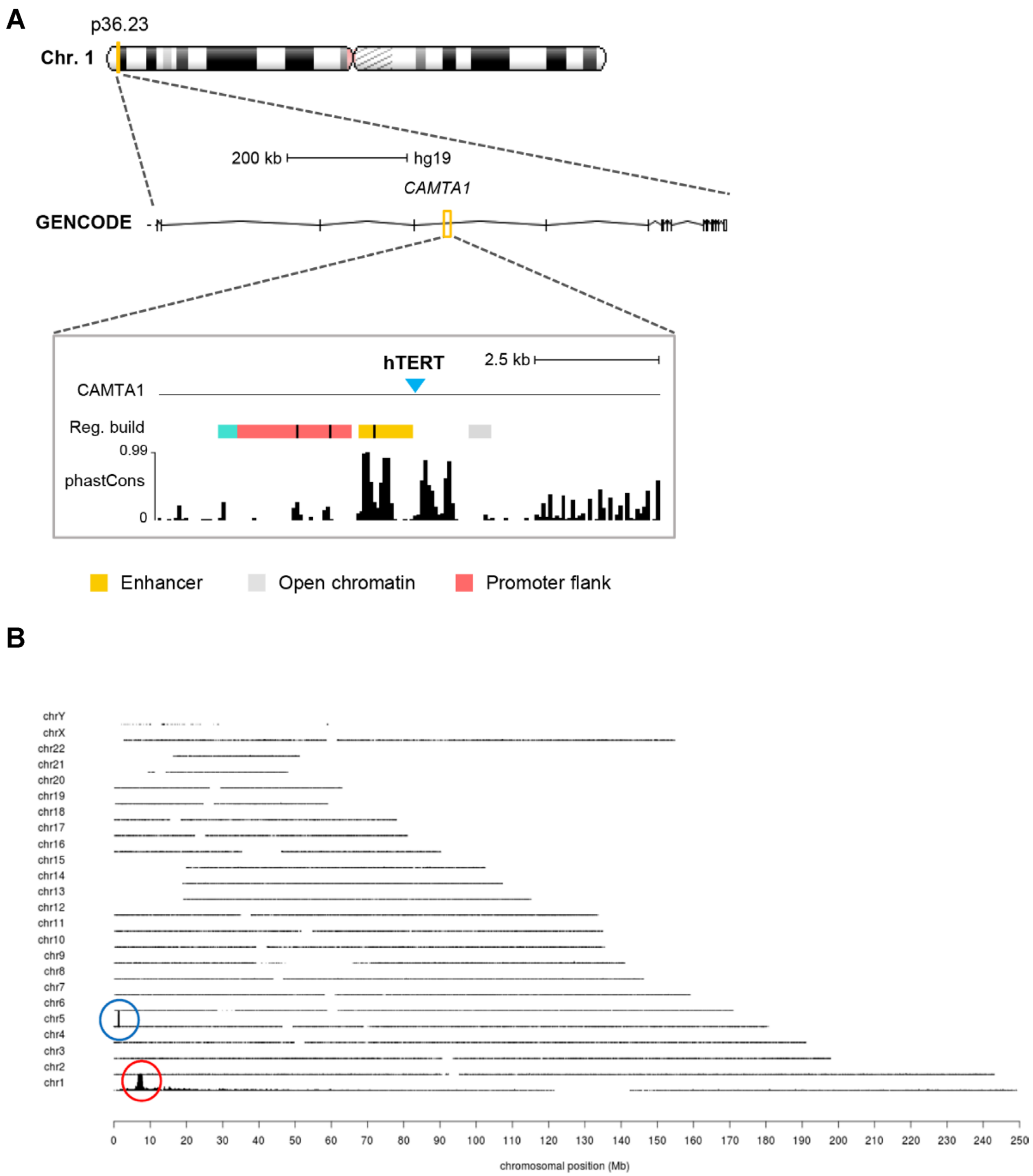

Supplementary Figure 3: Viral integration sites of *hTERT*. The integration sites of the gene encoding hTERT within (**A**) *CAMTA1*. The phastCons P100 database was used to identify evolutionary conserved regions. Gene legend: untranslated region (empty box), exon (filled box), intron (line). (**B**) TLA sequence coverage across the human genome with different chromosomes indicated on the y-axis, and the chromosomal position on the x-axis. The position of the integration site of *hTERT* is encircled in red. Encircled in blue is the position of hTERT gene locus.

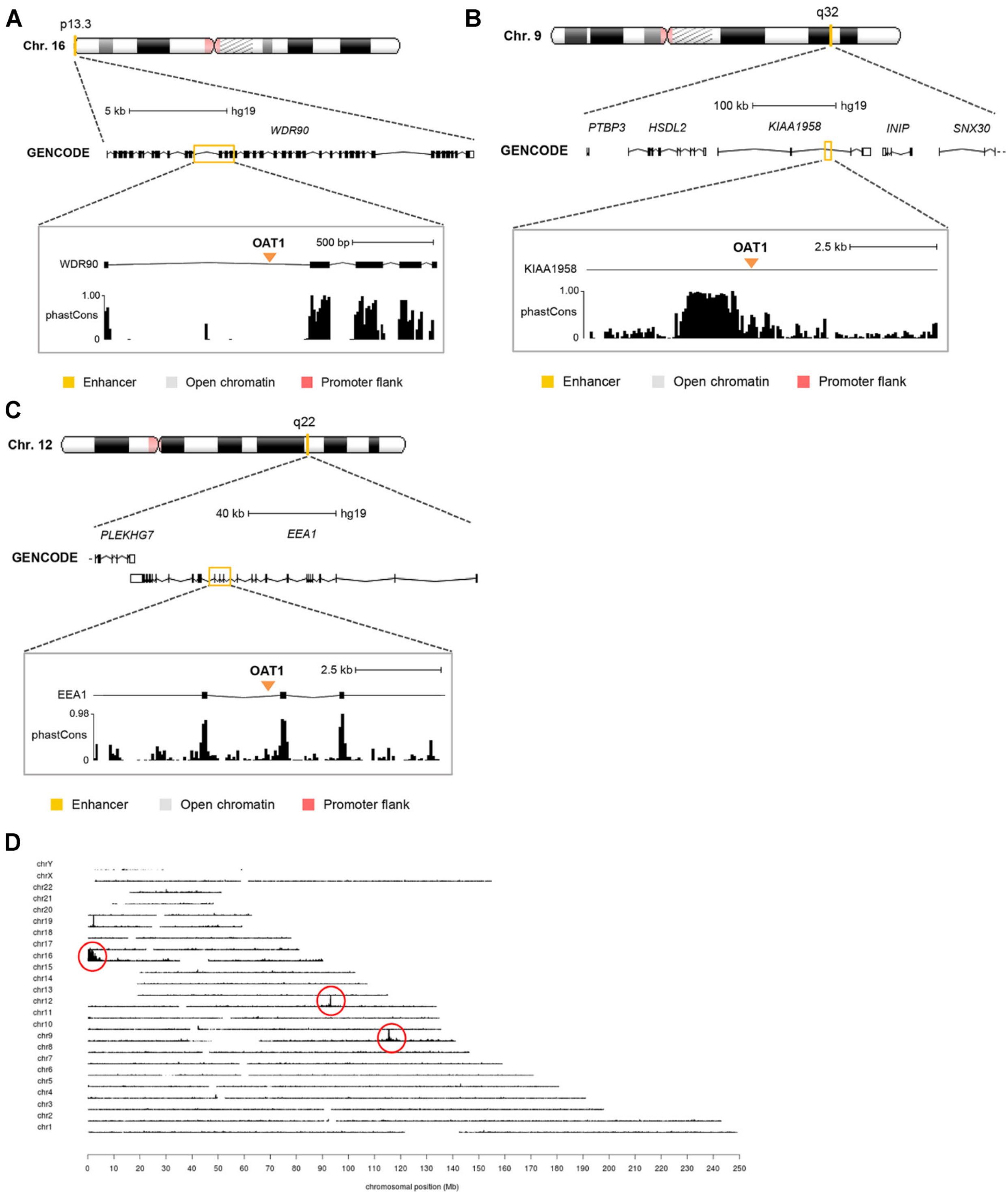

Supplementary Figure 4: Viral integration sites of *SLC22A6* (encoding OAT1). The integration sites of the gene encoding OAT1 within (**A**) *WDR90*, (**B**) *KIAA1958* and (**C**) *EEA1*. The phastCons P100 database was used to identify evolutionary conserved regions. Gene legend: untranslated region (empty box), exon (filled box), intron (line). (**D**) TLA sequence coverage across the human genome with different chromosomes indicated on the y-axis, and the chromosomal position on the x-axis. The position of the integration sites of the OAT1-encoding gene are encircled in red.

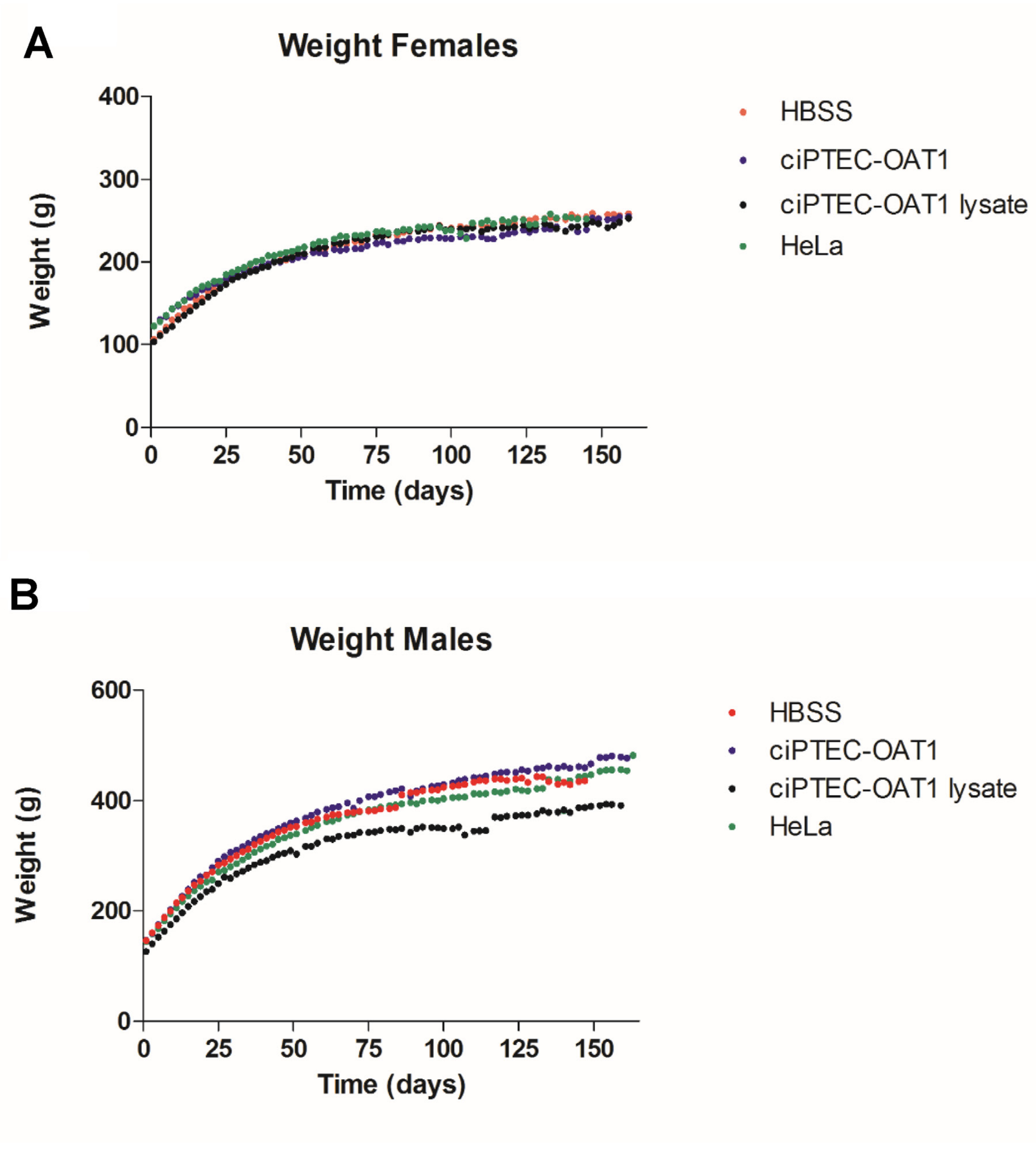

Supplementary Figure 5: Weight measurements of rats for 5 months study. Weight measurements (grams; g) in (**A**) female and (**B**) male rats during the follow-up.
